# Upregulation of the histone γ-H2AX correlates with worse patient survival and basal-like subtype in pancreatic ductal adenocarcinoma

**DOI:** 10.1007/s00432-024-05681-x

**Published:** 2024-03-19

**Authors:** Karl Knipper, Yussra Hussein, Adrian Georg Simon, Caroline Fretter, Alexander I. Damanakis, Yue Zhao, Christiane J. Bruns, Thomas Schmidt, Felix C. Popp, Alexander Quaas, Su Ir Lyu, The PANCALYZE Study Group, Michael Heise, Frank Marusch, Marco Siech, Tawfik Mosa, Bodo Schniewind, Jürgen Tepel, Werner Hartwig, Christoph Prinz, Bettina M. Rau, Marco Niedergethmann, Rainer Kube, George Saada, Wolfgang Hiller, Utz Settmacher

**Affiliations:** 1 Faculty of Medicine and University Hospital of Cologne, Department of General, Visceral and Cancer Surgery, University of Cologne, Cologne, Germany; 2 Faculty of Medicine and University Hospital of Cologne, Institute of Pathology, University of Cologne, Cologne, Germany

**Keywords:** Pancreatic ductal adenocarcinoma, H2AX, Histone, DNA repair, Biomarker

## Abstract

**Purpose:**

Patients with pancreatic ductal adenocarcinoma (PDAC) have yet to experience significant benefits from targeted therapy. Olaparib is currently the only active substance in BRCA-mutated PDACs that successfully influences the DNA repair of carcinoma cells. H2AX belongs to the histone family and is known as a part of the DNA repair system. The inhibition of γ-H2AX could lead to the inhibition of mitotically active tumor cells. Therefore, we aimed to evaluate the predictive value of the γ-H2AX in patients with PDAC.

**Methods:**

All included patients (*n* = 311) received a pancreatic resection with curative intention in one of our PANCALYZE study centers. Subsequently, they were enrolled in a standardized follow-up protocol. Immunohistochemical stainings for γ-H2AX were conducted on tissue microarrays.

**Results:**

Patients exhibiting high levels of γ-H2AX expression experience more frequent R1 resections, indicating advanced tumor stages in this subgroup. Additionally, patients with high γ-H2AX expression demonstrated significantly poorer survival compared to those with low expression (median OS: 15 vs. 25 months, *p* < 0.001). In multivariate analyses, high γ-H2AX expression could be identified as an independent risk factor for worse patient survival. Moreover, high γ-H2AX expression could be more frequently observed in the more aggressive basal-like subtype.

**Conclusion:**

γ-H2AX can be characterized as a predictive biomarker for poorer patient survival. Consequently, upcoming clinical trials focused on the efficacy of targeted therapies influencing the DNA repair system and radiotherapy should evaluate γ-H2AX as a potential biomarker for therapy response. Furthermore, γ-H2AX may serve as a viable target for treatment in the future.

## Introduction

Despite recent advancements in first-line therapy for pancreatic ductal adenocarcinoma (PDAC) leading to modest improvements in survival, the discovery of more effective treatments for these patients remains an ongoing challenge (Fang et al. 2023; Conroy et al. 2018). Long-term survival following resection and adjuvant chemotherapy is still uncommon (Goor et al. 2024). Despite significant advancements in targeted therapy for other solid malignancies, the much anticipated breakthrough in targeted therapy for patients with pancreatic ductal adenocarcinoma (PDAC) is yet to be achieved. (Fang et al. 2023; Majeed et al. 2021; Lorentzen 2019). For instance, newly discovered treatment options influencing the DNA repair system are evolving, especially in breast cancer patients. Here, patients with BRCA1 and BRCA2 mutations are treated with poly(ADP-ribose) polymerase (PARP) inhibitors. Following this, cancer cells are no longer able to perform DNA repair, leading to cell cycle arrest. With this therapy, the progression-free survival of patients with breast cancer could be significantly improved (Cortesi et al. 2021). Whole genome analyses revealed that mutations in DNA maintenance genes also occur in the pancreatic ductal adenocarcinomas (Waddell et al. 2015). Therefore, PARP inhibitors, among other treatments, are currently being investigated for their potential efficacy in these patients (Keane et al. 2023). A recent phase 3 trial explored the use of olaparib as maintenance therapy for patients with BRCA1 or BRCA2 mutations and metastatic pancreatic cancer. In this study, patients treated with olaparib demonstrated a significant improvement in progression-free survival compared to those receiving placebo (7.4 months vs. 3.8 months). However, no change in overall survival was noted (Golan et al. 2019). Notably, patients treated with olaparib more frequently achieved long-term survival and experienced a significantly longer duration before requiring subsequent chemotherapy (Kindler et al. 2022). This indicates the possibility of the existence of a subgroup of patients with PDAC who benefit from PARP inhibitors or hypothetically other inhibitors of the DNA repair system.

H2AX belongs to the histone family and is known as a part of the DNA repair system (Kuo and Yang 2008; Foster and Downs 2005; Ausio and Abbott 2002). Within minutes of DNA damage, H2AX undergoes phosphorylation to become γ-H2AX (Rogakou et al. 1998). These phosphorylated proteins accumulate around the damaged area, subsequently attracting a multitude of well-known DNA repair factors like BRCA1 (Paull et al. 2000; Fernandez-Capetillo et al. 2003). Therefore, γ-H2AX is recognized as a vital component in the initiation of effective DNA repair systems. Moreover, the inhibition of H2AX could hypothetically lead to the inhibition of highly mitotic cells, such as cancer cells, thereby contributing to disease control. Indeed, H2AX-defective mice showed growth retardation, immune deficiency, and male infertility (Celeste et al. 2002). γ-H2AX is highly upregulated in cancer cells compared to the corresponding normal tissue (Sedelnikova and Bonner 2006).

In this study, our objective was to elucidate the impact of γ-H2AX expression in pancreatic ductal adenocarcinoma.

## Materials and methods

### Patients and tumor samples

All the included patients were diagnosed and resected at one of the mentioned PANCALYZE study centers. The pancreatic resections were performed with curative intention between the years 2012 and 2020. Patients with a shorter survival period than 30 postoperative days or insufficient tumor sample quality were excluded. All data were collected prospectively as defined in the PANCALYZE study protocol and analyzed retrospectively (Popp et al. 2017). Tumor samples and patient data were transferred to the study center (University Hospital of Cologne). Written informed consent was obtained from every patient. The study was approved by the Ethics Committee of the University Hospital of Cologne (ethics committee number: 16-230) and was conducted following the Declaration of Helsinki. The tumor stage values were assessed following the 7th edition of the Union for International Cancer Control.

The received tumor samples were evaluated regarding tissue quality by an experienced pathologist. Furthermore, tumor borders were marked and two 1.2-ml tissue cylinders were punched out with a semi-automated precision instrument. These cylinders were transferred to a paraffin-embedded tissue microarray. Further experiments were conducted on 4-µm-thick slices of the microarray.

### Immunohistochemistry

Immunohistochemical stainings were performed with the automatic staining system Leica BOND-MAX (Leica Biosystems, Wetzlar, Germany). γ-H2AX stainings were conducted according to the manufacturer (γ-H2AX: ab81299, Abcam, Cambridge, UK, 1:200, EDTA, control: breast carcinoma). The analyses were carried out by two experienced pathologists (AQ and SL). γ-H2AX stainings were assessed for the intensity of staining and percentage of stained tumor cells. Hereafter, the *H*-Score was calculated for each tumor sample (Mazieres et al. 2013). The mean of both tumor samples of each patient was used for the ongoing analyses. The mean of the H-score of all included patients was 120. We divided our study cohort into patients with low (*H*-score < 120) and high γ-H2AX expression levels (*H*-score ≥ 120) by the mean.

### Statistical analysis

IBM SPSS Statistics (Version 29.0.1.1, Armonk, USA) was used for all statistical analyses. *p* values below 0.05 were considered statistically significant. Survival analyses were conducted with Kaplan–Meier curves and log-rank tests. Qualitative values were related using the Chi-square test. Overall survival was defined as the time between resection and patients’ death or loss of follow-up. In contrast, disease-free survival was defined as the time between resection and diagnosis of disease recurrence of any kind or loss of follow-up. Interdependencies between clinicopathologic values and overall survival were calculated with univariate and multivariate Cox regression analyses. Only clinicopathological values with a *p* value below 0.2 in the univariate Cox regression analysis were included in the multivariate Cox regression analysis.

## Results

All 311 patients included in the study were diagnosed with pancreatic ductal adenocarcinoma (Table 1). The sex distribution was almost equal in our total cohort (male: 49.5%, female: 50.5). The median survival for all patients included in the study was 17.0 months. 4.2% (*n* = 13) were treated with neoadjuvant chemotherapy. Most of the included patients were diagnosed with lymph node metastases (72.0%). Complete tumor resection could be achieved in approximately two-thirds of the study cohort (64.3%).

**Table 1:** General clinicopathological values of the total study population and patients with low or high γ-H2AX expression

Characteristic	Total	γ-H2AX		*p*-value
		Low	High	
	*n* (%)	*n* (%)	*n* (%)	
No. of patients	311 (100)	175 (100)	136 (100)	
Sex				0.759
Male	154 (49.5)	88 (50.3)	66 (48.5)	
Female	157 (50.5)	87 (49.7)	70 (51.5)	
Age				0.399
< 65	97 (31.2)	58 (33.1)	39 (28.7)	
≥ 65	214 (68.8)	117 (66.9)	97 (71.3)	
Median overall survival (months)	17.0 (2.0–98.0)	21.0 (2.0–98.0)	14.0 (2.0–72.0)	0.453
Neoadjuvant therapy				
No	299 (96.1)	167 (95.4)	132 (97.1)	
Yes	12 (3.9)	8 (4.6)	4 (2.9)	
(y)pT				0.076
1	21 (6.8)	14 (8.0)	7 (5.1)	
2	114 (36.7)	72 (41.1)	42 (30.9)	
3	169 (54.3)	87 (49.7)	82 (60.3)	
4	7 (2.3)	2 (1.1)	5 (3.7)	
(y)pN				0.603
0	87 (28.0)	51 (29.1)	36 (26.5)	
1	224 (72.0)	124 (70.9)	100 (73.5)	
R				** < 0.001**
0	200 (64.3)	128 (73.1)	72 (52.9)	
1	110 (35.4)	46 (26.3)	64 (47.1)	
2	1 (0.3)	1 (0.6)	0 (0.0)	
L				0.322
0	119 (38.3)	71 (40.6)	48 (35.3)	
1	191 (61.4)	103 (58.9)	88 (64.7)	
Unknown	1 (0.3)	1 (0.6)	0 (0.0)	
V				0.428
0	214 (68.8)	123 (70.3)	91 (66.9)	
1	93 (29.9)	48 (27.4)	45 (33.1)	
Unknown	4 (1.3)	4 (2.3)	0 (0.0)	
pN				0.486
0	74 (23.8)	45 (25.7)	29 (21.3)	
1	226 (72.7)	127 (72.6)	99 (72.8)	
Unknown	11 (3.5)	3 (1.7)	8 (5.9)	
G				0.122
1	8 (2.6)	5 (2.9)	3 (2.2)	
2	152 (48.9)	95 (54.3)	57 (41.9)	
3	136 (43.7)	65 (37.1)	71 (52.2)	
4	3 (0.9)	2 (1.1)	1 (0.7)	
Not applicable/unknown	12 (3.9)	8 (4.6)	4 (2.9)	

Bold print marks *p* values below 0.05

**Fig. 1:**
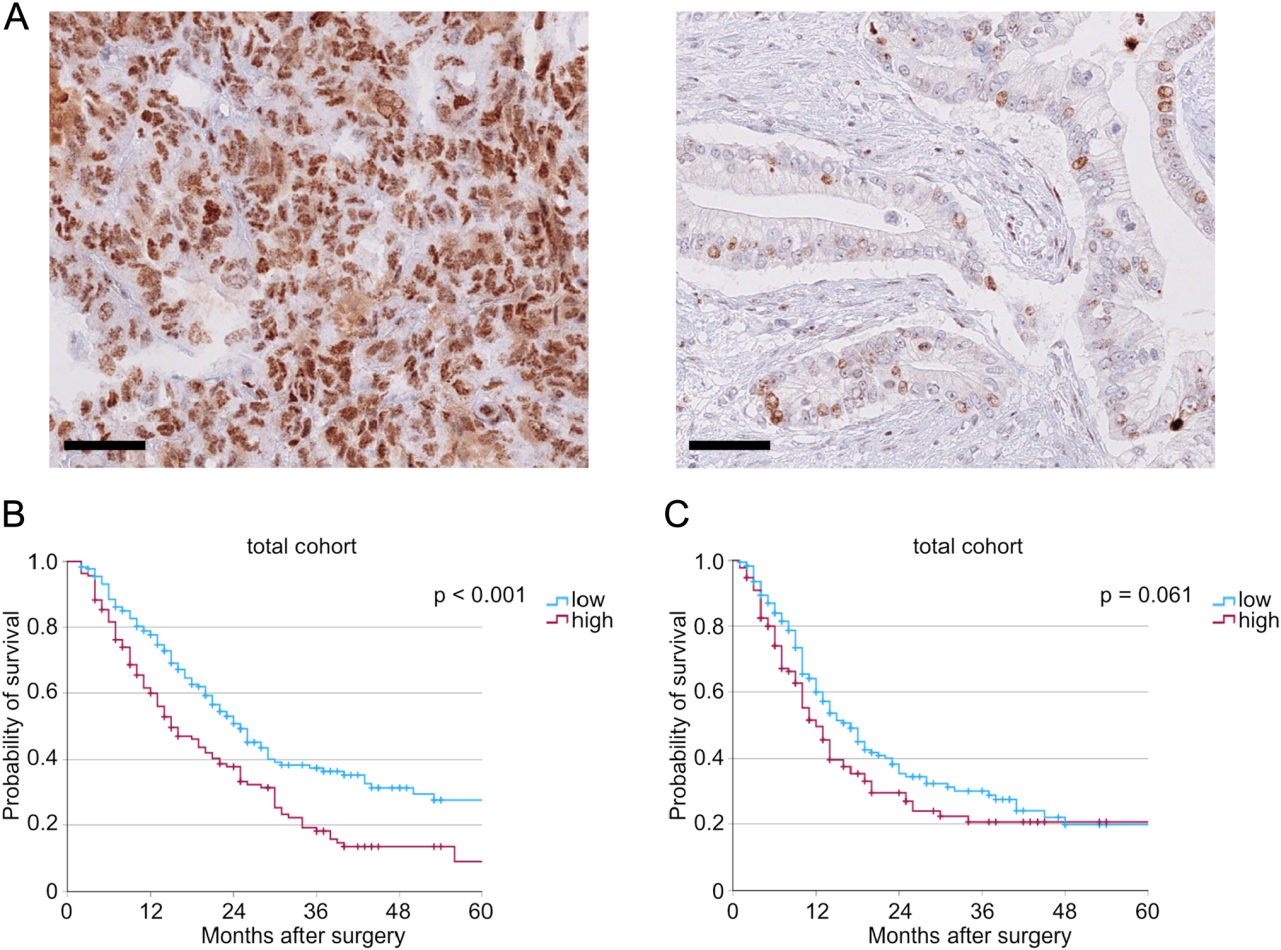
**A** Exemplary pictures of tumors with high (left) and low (right) γ-H2AX expression. **B** Kaplan–Meier curve for overall survival (*n*(low) = 175, *n*(high) = 136, *p* < 0.001) and (**C**) for disease-free survival depending on γ-H2AX expression levels of the total cohort (*n*(low) = 173, *n*(high) = 132, *p* = 0.061). Side bar: 50 µm

Immunohistochemical stainings for γ-H2AX were performed and analyzed. All PDACs showed positivity for γ-H2AX at different expression intensities. 175 tumors displayed weak γ-H2AX expression (56.3%), while 136 tumors showed high tumor cell expression of γ-H2AX (43.7%). Exemplary images showcasing tumors with high and low γ-H2AX expression are presented in Fig. 1 A. We compared widely used clinicopathologic valuables between these two groups. While no significant differences in T- and N-stages with a trend to higher T-stages in patients with high γ-H2AX expression (*p* = 0.076) could be observed, patients with high γ-H2AX expression showed significantly more often a microscopically stated incomplete resection.

Furthermore, we conducted survival analyses to assess the impact of γ-H2AX expression on patients’ outcomes. Here, patients with high γ-H2AX expression showed significantly worse overall survival compared to patients with low γ-H2AX expression (median OS: 15 vs. 25 months, *p* < 0.001, Fig. 1B). Similar analyses were performed for disease-free survival. No significant differences between patients with high and low γ-H2AX expression in disease-free survival could be detected (median OS: 12 vs. 17 months, *p* = 0.061, Fig. 1C).

To assess possible interdependencies between clinicopathologic values and patient outcomes, we proceeded with univariate and multivariate Cox regression analyses. Here, high γ-H2AX expression could be described as a significant independent risk factor for worse patient outcome (HR = 1.600, 95% CI 1.202–2.130, *p* = 0.001, Table 2). Additionally, higher (y)pT-stage, higher (y)pN-stage, and higher G-status proved to be significant factors for worse patient overall survival ((y)pT: HR = 1.287, 95% CI 1.008–1.645, *p* = 0.043; (y)pN: HR = 2.381, 95% CI 1.612–3.516, *p* < 0.001; G: HR = 1.283, 95% CI 1.007–1.635, *p* = 0.043, Table 2).

**Table 2:** Multivariate Cox regression analyses of the total study cohort

Characteristic	Borders	Hazard ratio	95% confidence interval	*p* value
(y)pT	≥ 2 vs 1	1.287	1.008–1.645	**0.043**
(y)pN	≥ 1 vs 0	2.381	1.612–3.516	** < 0.001**
R	1 vs 0	1.210	0.911–1.607	0.188
L	1 vs 0	0.764	0.544–1.074	0.121
pN	≥ 1 vs 0	0.989	0.674–1.452	0.955
G	≥ 2 vs 1	1.283	1.007–1.635	**0.043**
γ-H2AX	High vs low	1.600	1.202–2.130	**0.001**

Bold print marks *p* values below 0.05

Since γ-H2AX was described to be accumulated in patients with a basal-like subtype of breast cancer, we compared the distribution of the basal-like subtype in tumors with low and high γ-H2AX expression levels (Nagelkerke et al. 2011). The definition of the basal-like subtype in the described patient cohort has been published by our group before (Lyu et al. 2023). Here, we could show that tumors with high γ-H2AX expression exhibit significantly more often a basal-like subtype (*p* < 0.001, Table 3).

**Table 3:** The distribution of the basal-like subtype depending on low or high γ-H2AX expression is depicted

Characteristic	γ-H2AX		*p* value
	Low	High	
	*n* (%)	*n* (%)	
No. of patients	168 (100)	130 (100)	
Basal-like subtype			** < 0.001**
No	125 (74.4)	70 (53.8)	
Yes	43 (25.6)	60 (45.3)	

Bold print marks *p* values below 0.05

In summary, we describe high γ-H2AX expression as an independent factor for worse patient survival in a patient cohort with pancreatic ductal adenocarcinoma.

## Discussion

This study, which is a part of the multi-center PANCALYZE study, investigated the role of γ-H2AX in pancreatic ductal adenocarcinoma. Thus, we performed immunohistochemical stainings for γ-H2AX in 311 primary tumors. In our findings, we observed that a high γ-H2AX expression is correlated with worse patient survival. Furthermore, a high γ-H2AX expression proved to be an independent risk factor for poor prognosis (HR = 1.600, 95% CI 1.202–2.130, *p* = 0.001). These findings are supported by previously published studies from different solid malignancies. High γ-H2AX expression was associated with worse overall survival in colorectal cancer and non-small cell lung cancer (Lee et al. 2015; Matthaios et al. 2012). Moreover, high γ-H2AX expression was correlated with higher tumor stage and perineural invasion in patients suffering from colorectal cancer (Lee et al. 2015). In our study cohort, we could only find a trend toward higher tumor stages in tumors with high γ-H2AX expression (*p* = 0.076). However, high γ-H2AX expression correlated with a higher rate of microscopic incomplete resections (*p* < 0.001). This indicates the possibility that tumors exhibiting high levels of γ-H2AX expression are likely to have a more invasive growth pattern and, indirectly, more advanced tumor stages.

However, γ-H2AX does not only correlate with worse patient survival and higher tumor stages, but also with specific tumor subtypes. In breast cancer, positive γ-H2AX expression was correlated with the basal-like phenotype (Nagelkerke et al. 2011). The basal-like tumor subtype is described in breast cancer, but also in pancreatic ductal adenocarcinoma (Moffitt et al. 2015; Perou et al. 2000). The basal-like subtype is compared to the classical subtype known to be correlated with poor prognosis for both tumor entities (Lyu et al. 2023; Nielsen et al. 2004; O'Kane et al. 2020). We compared the distribution of the basal-like subtype in our patient cohort depending on the γ-H2AX expression based on the previously published definition of the basal-like subtype of our study group (Lyu et al. 2023). Indeed, significantly more basal-like tumors could be found in the subgroup of tumors with high γ-H2AX expression (*p* < 0.001). In summary, γ-H2AX expression is not only correlated with poor prognosis itself but also with the pre-described basal-like subtype.

γ-H2AX could also be used as a biomarker in blood samples and could therefore facilitate diagnostics of patients during screening or follow-up exams since blood samples could be assembled non-invasively. The γ-H2AX assay is a diagnostic tool that has been established in previous studies (Xu et al. 2013). Here, peripheral blood lymphocytes are treated with irradiation. The γ-H2AX levels are measured before (baseline) and after irradiation (irradiation induced). Following this, the ratio of irradiation-induced and baseline γ-H2AX levels are calculated. The ratios of patients with bladder cancer or esophageal adenocarcinoma were significantly higher compared to healthy controls. Furthermore, higher ratios could be associated with a higher risk of the occurrence of esophageal adenocarcinoma or bladder cancer (Xu et al. 2013; Fernandez et al. 2013). Unfortunately, the PANCALYZE study does not collect peripheral blood samples. Considering our findings that highlight the significant role of γ-H2AX in patients with pancreatic ductal adenocarcinoma (PDAC), future research should explore the potential of γ-H2AX as a non-invasive biomarker in peripheral blood.

Radiotherapy solely or combined radiochemotherapy is not widely recommended as a treatment strategy for patients with PDAC (Conroy et al. 2023). For instance, a recent study that compared preoperative mFOLFIRINOX with mFOLFIRINOX plus radiotherapy in patients with borderline resectable adenocarcinoma reported a decrease in overall survival for those who received radiochemotherapy (Katz et al. 2022). These results contrast with results in other malignancies for example esophageal cancer (Obermannova et al. 2022). Hypothetically, the biomarkers currently in use are unable to differentiate between PDAC patients who might benefit from radiotherapy and those who exhibit reduced radiosensitivity. Assessing the potential response to therapy before initiating treatment is a key objective of personalized medicine. This approach could help avoid adverse events from treatments that do not enhance patient outcomes. A retrospective study of patients with colorectal adenocarcinoma could show, that higher γ-H2AX expression levels in the resected specimen could be detected within the tumors with low treatment response following chemoradiotherapy compared to tumors with high regression (Kawashima et al. 2020). This suggests that patients with low γ-H2AX expression levels in their tumor cells might derive greater benefit from chemoradiotherapy. To further explore this concept, it may be possible in the future to assess γ-H2AX expression in primary biopsies of PDAC patients, which could then influence the selection of neoadjuvant (radio-)chemotherapy regimens.

As our research suggests, γ-H2AX could serve as a biomarker following further prospective studies. Additionally, it may also be considered as a potential target for treatment. Interventions targeting pathways that alter H2AX expression could be demonstrated to influence the survival of tumor cells. The treatment of gastrointestinal stromal tumors with imatinib, a widely used selective small-molecule protein kinase inhibitor, results in an upregulation of soluble H2AX. A depletion of H2AX results in an inhibited apoptotic effect of imatinib on the tumor cells (Liu et al. 2007). Conversely, the efficacy of radiotherapy could be enhanced by inhibiting γ-H2AX, thereby increasing the radiosensitivity (Kao et al. 2006). However, the regulation of γ-H2AX is complex, and interference in this system depends not only on the specific target of intervention, but also on the timing of the intervention. Here, inhibition of protein phosphatase 2A, an enzyme involved in removing γ-H2AX from DNA break foci, can lead to inefficient DNA repair. This, in turn, results in an increased sensitivity to DNA damage, such as that caused by radiation (Chowdhury et al. 2005).

The retrospective nature of our study protocol is a limiting factor. Additionally, the detection of γ-H2AX has its limitations. The evaluation of γ-H2AX expression, particularly in blood samples, must take into account the physiological background level of γ-H2AX (Podhorecka et al. 2010). Furthermore, protein detection by immunohistochemistry is prone to error. Alterations of the protocol or different evaluators in various centers could lead to different results. These aspects must be meticulously assessed in future prospective study cohorts, which should include comparisons between healthy individuals and patients with PDAC with several protein detection methods. Contrary to our study cohort, Xu et al. reports that the administration of Cisplatin causes an increase of γ-H2AX in pancreatic carcinoma cells in vitro (Xu et al. 2022). In our study cohort, no significant increase of γ-H2AX expression in patients following neoadjuvant therapy could be observed. These competing results could be explained as Xu et al. conducted in vitro experiments on cultured pancreatic carcinoma cells. On the contrary, we report data based on immunohistochemical stainings in human tissue. Second, our database included in total only 12 patients (3.9%), who underwent neoadjuvant therapy before resection. This could be explained as neoadjuvant therapy was recently recommended for patients with borderline resectable or unresectable pancreatic tumors (Conroy et al. 2023). Therefore, further studies are needed to investigate the impact of neoadjuvant therapy on γ-H2AX expression in patients with PDAC.

In summary, our study positions γ-H2AX as a potential biomarker in patients with PDAC. We found that high γ-H2AX expression is associated with poorer patient survival, a finding corroborated by multivariate Cox regression analyses. Further prospective studies are required to delve deeper into the role of γ-H2AX in PDAC. Hypothetically, γ-H2AX could serve multiple purposes: as a biomarker for patient survival, as a criterion for initial treatment selection, as an indicator of therapy response, and possibly as a novel therapeutic target.

## Conclusions

We evaluated the predictive value of γ-H2AX expression in 311 patients with PDAC. Here, patients with high γ-H2AX expression showed a significantly worse overall survival. Given that γ-H2AX plays a crucial role in the physiological DNA repair system, we propose the evaluation of γ-H2AX expression as a potential biomarker for tumor response in future clinical trials of targeted treatments that interfere with the DNA repair system. Additionally, γ-H2AX could be used as a biomarker for survival, treatment strategy selection, and therapy response. Moreover, γ-H2AX should be evaluated as a potential treatment target.

## Data Availability

The datasets generated and analyzed during the current study are available from the corresponding author on reasonable request.
